# A New Freshwater Cyanosiphovirus Harboring Integrase

**DOI:** 10.3389/fmicb.2018.02204

**Published:** 2018-09-19

**Authors:** Kevin Xu Zhong, Curtis A. Suttle, Anne-Claire Baudoux, Evelyne Derelle, Jonathan Colombet, Anna Cho, Jessica Caleta, Christophe Six, Stéphan Jacquet

**Affiliations:** ^1^INRA, UMR 042 CARRTEL, Thonon-les-Bains, France; ^2^Department of Earth, Ocean, and Atmospheric Sciences, University of British Columbia, Vancouver, BC, Canada; ^3^Department of Microbiology and Immunology, University of British Columbia, Vancouver, BC, Canada; ^4^Department of Botany, Institute for Oceans and Fisheries, University of British Columbia, Vancouver, BC, Canada; ^5^Sorbonne Universités UPMC Paris 06, CNRS, UMR7144 Adaptation et Diversité en Milieu Marin, Station Biologique de Roscoff, Roscoff, France; ^6^Integrative Marine Biology Laboratory (BIOM), CNRS UMR7232, Sorbonne Universities, Banyuls-sur-Mer, France; ^7^CNRS, Université Blaise Pascal, UMR 6023, Laboratory of Microorganismes, Aubière, France

**Keywords:** freshwater, lakes, *Synechococcus*, cyanosiphovirus, genome sequencing

## Abstract

Pelagic cyanobacteria are key players in the functioning of aquatic ecosystems, and their viruses (cyanophages) potentially affect the abundance and composition of cyanobacterial communities. Yet, there are few well-described freshwater cyanophages relative to their marine counterparts, and in general, few cyanosiphoviruses (family *Siphoviridae*) have been characterized, limiting our understanding of the biology and the ecology of this prominent group of viruses. Here, we characterize S-LBS1, a freshwater siphovirus lytic to a phycoerythrin-rich *Synechococcus* isolate (Strain TCC793). S-LBS1 has a narrow host range, a burst size of ∼400 and a relatively long infecting step before cell lysis occurs. It has a dsDNA 34,641 bp genome with putative genes for structure, DNA packing, lysis, replication, host interactions, DNA repair and metabolism. S-LBS1 is similar in genome size, genome architecture, and gene content, to previously described marine siphoviruses also infecting PE-rich *Synechococcus*, e.g., S-CBS1 and S-CBS3. However, unlike other *Synechococcus* phages, S-LBS1 encodes an integrase, suggesting its ability to establish lysogenic relationships with its host. Sequence recruitment from viral metagenomic data showed that S-LBS1-like viruses are diversely present in a wide range of aquatic environments, emphasizing their potential importance in controlling and structuring *Synechococcus* populations. A comparative analysis with 16 available sequenced cyanosiphoviruses reveals the absence of core genes within the genomes, suggesting high degree of genetic variability in siphoviruses infecting cyanobacteria. It is likely that cyanosiphoviruses have evolved as distinct evolutionary lineages and that adaptive co-evolution occurred between these viruses and their hosts (i.e., *Synechococcus, Prochlorococcus*, *Nodularia*, and *Acaryochloris*), constituting an important driving force for such phage diversification.

## Introduction

Cyanobacteria are often the most abundant and widely distributed autotrophs in aquatic environments, where they play major roles in carbon fixation and trophic interactions. Especially, members of the genus *Synechococcus* are among the most ubiquitous and abundant picophytoplankters in fresh and marine waters (Partensky et al., 1999; Callieri and Stockner, 2002). During the last three decades, studies have shown that viruses infecting cyanobacteria (cyanophages) can be major players affecting the mortality, dynamics, diversity, and structure of cyanobacterial communities (reviewed in Suttle, 2000, 2007; Mann, 2003; etc.).

To study cyanophage diversity, PCR- and metagenomic-based methods were developed in recent years. PCR-based approaches require primers to target specific genes associated with specific groups of viruses. However, such primers are based on the limited number of available sequences in databases, leading to bias (Zhong and Jacquet, 2013). Moreover, most viral sequences in metagenomic databases still have no putative function (Hurwitz and Sullivan, 2013), and a first step in assigning function is to place them into a genomic context (Chow et al., 2015). This is particularly true for fresh waters, for which, to the best of our knowledge, only two virus isolates infecting *Synechococcus* (i.e., S-CM01 and S-EIV1) have been sequenced (Dreher et al., 2011; Chénard et al., 2015).

About half a century ago, the first cyanophages infecting freshwater filamentous cyanobacteria were isolated (Safferman and Morris, 1963). Subsequently, several freshwater cyanophages were isolated (Safferman et al., 1969; Safferman et al., 1972; Stewart and Daft, 1977), but knowledge on their ecology remained scarce. Cyanophages infecting marine cyanobacteria were isolated in the 1990’s (Suttle and Chan, 1993; Waterbury and Valois, 1993) but it was a decade later that the first genome of a cyanophage (e.g., P60) infecting the marine picophytoplankter *Synechococcus* was reported (Chen and Lu, 2002).

Cyanophages are tailed dsDNA viruses within the order Caudovirales, and consist of three families (*Myoviridae*, *Podoviridae*, and *Siphoviridae*) based on having either a long contractile tail, a short non-contractile tail, or a long flexible tail, respectively (Safferman et al., 1983). They are colloquially referred to as cyanomyoviruses, cyanopodoviruses or cyanosiphoviruses, respectively (Suttle, 2000). The large majority of phages infecting *Synechococcus* spp. isolates are myoviruses but podoviruses have also been isolated and genetically characterized (Dekel-Bird et al., 2013; Labrie et al., 2013). Information on cyanosiphoviruses, especially genomic characterization, are rare and exclusively marine (Sullivan et al., 2009; Huang et al., 2012; Mizuno et al., 2013; Ponsero et al., 2013; Chan et al., 2015; Coloma et al., 2017). These cyanosiphoviruses are suspected or reported to infect picocyanobacteria (*Prochlorococcus* spp. or *Synechococcus* spp.), the facultative epibiont *Acaryochloris marina* and the filamentous nitrogen fixer *Nodularia* sp.

Cyanophages in aquatic systems have been comprehensively reviewed by several occasions (e.g., Suttle, 2000, 2007; Wommack and Colwell, 2000; Mann, 2003; etc.). In freshwater ecosystems, a worldwide distribution of cyanophages infecting bloom-forming species has been reported with concentrations ranging typically from 10^2^ to 10^5^ particles mL^-1^. Viruses infecting *Synechococcus* isolates are more specifically related to phycocyanin (PC)-rich strains in freshwater and to phycoerythrin (PE)-rich strains in marine ecosystems (Suttle, 2000; Mann, 2003). Although PE-rich strains can be dominant in many freshwater systems (Caron et al., 1985; Stockner et al., 2000; Personnic et al., 2009), only a few attempts to isolate viruses infecting these cyanobacteria are described in the literature (Suttle and Chan, 1993; Waterbury and Valois, 1993). In marine ecosystems, most efforts were deployed to isolate viruses infecting single strains of unicellular *Synechococcus* spp. (Suttle, 2000; Mann, 2003).

Our study expands on reports highlighting the high diversity of cyanophages in peri-alpine lakes (Dorigo et al., 2004; Zhong and Jacquet, 2013). These phages may cause, at some periods of the year, significant mortality of the *Synechococcus* spp. community (Personnic et al., 2009; Zhong et al., 2013). Attempts to isolate cyanophages on PE-rich picocyanobacteria isolated from French-Alpine lakes led to the discovery of the cyanosiphovirus described in this article.

## Materials and Methods

### Study Site

Lake Bourget (45°44′N, 05°52′E, 231 m elevation) is the largest natural deep lake in France, located at the edge of the Alps. It is elongated with a length and width of 18 and 3.5 km, respectively, and is north-south orientated. It is characterized by an area of 44 km^2^, total volume of 3.5 km^3^, a maximal and average depth of 145 and 80 m, respectively, and a water residence time of about 10 years. This lake was scientifically surveyed and sampled every 3–4 weeks since 2004 (Jacquet et al., 2014).

### *Synechococcus* Host Isolation and Characterization

#### Isolation

In 2011, *Synechococcus* sp. TCC793 was isolated, during the year from the 10 m depth of Lake Bourget at the reference station in the middle and deepest part of the lake, using the sorting function of a FACSCalibur flow cytometer (FCM), detailed in **Supplementary Methods**. This isolated *Synechococcus* was then characterized by measuring the cell size using microscope, identifying pigments using spectrofluorimeter and high pressure liquid chromatography (HPLC). Technical details of these methods can be found in **Supplementary Methods**.

#### Bacterial Genomic DNA Extraction, PCR, Cloning and Sequencing

Cyanobacterial genomic DNA was extracted from 0.2-μm pore-size polycarbonate filters using phenol-chloroform (Steward and Culley, 2010), and resuspended in TE buffer. A fragment of the 16S rRNA gene was amplified in a 50 μl PCR reaction using 1 μl of the resuspended genomic DNA mixed with 200 μM dNTP, 2 mM MgCl_2_, 0.12 mg/ml BSA, 10 pmol of the cyanobacteria-specific primers (CYA 371F: CCTACGGGAGGCAGCAGTGGGGAATTTTCCAC and 373R: CTAACCACCTGAGCTAAT), 1.5 U BioTaq. Optimal PCR conditions were as follows: an initial step at 94°C for 5 min, followed by 25 cycles at 94°C for 1 min, 60°C for 1 min, 72°C for 1 min, and a final step at 72°C for 5 min (BIOMETRA 070-601 thermocycler). The 1500 nt amplicons were purified using a GE Healthcare illustra GFX PCR DNA and Gel Band Purification Kit, and cloned into a plasmid using the Topo TA Cloning Kit, following supplier recommendations. Monoclonal amplicons were sent to GATC Biotech^TM^ (Germany) for sequencing. The ribosomal RNA gene sequence of *Synechococcus* sp. TCC793 was deposited in Genbank under accession number MG605056.

### Virus Isolation and Characterization

#### Infection Procedure

To isolate viruses infecting the purified PE-rich picocyanobacteria, water samples were collected during 2011 at the same depth of 10 m. Each time, 20 l of lake water was sampled using an electric pump on the boat deck. The water was filtered through glass-fiber GF/F filters (Whatman) followed by a 0.2 μm pore-size polycarbonate filter to remove the cellular fraction. The virus size fraction in the filtrate was concentrated 10- to 100-fold by tangential flow filtration using a mini-ultrasette with a 100 kDa cut-off membrane (Vivaflow, Vivasciences), and stored at 4°C before use. For infection experiments, all viral concentrates were pooled together and 5 ml of this pooled viral concentrate was added to 100 ml cultures of each strain (see below). After four rounds of infection and purification using an extinct-dilution method (Suttle, 1993), a lytic phage (S-LBS1) was isolated for *Synechococcus* strain TCC793.

#### Transmission Electron Microscopy

Viral morphology and burst size (BS) were determined by transmission electron microscopy (TEM) as described in Pradeep-Ram et al. (2011). Briefly, viruses and cells were diluted in 0.002-mm filtered-distilled-deionised water and collected onto a 400-mesh Cu electron microscopy grid supported with carbon-coated Formvar film (Pelanne Instruments, Toulouse, France) by ultracentrifugation at 70,000 × *g* for 20 min at 4°C using a SW40Ti rotor in a Beckman LE-80K ultracentrifuge (Brea, CA, United States). Each grid was stained at room temperature for 30 s with uranyl acetate (2% w/w), rinsed twice with distilled water, and dried on a filter paper. Grids were examined using a JEM 1200EX TEM (JEOL, Akishima, Japan) operated at 80 kV at a magnification of 25,000- to 200,000-fold. Individual virus morphotypes were identified, and measured using Axio Vision v4.7.1.0 (2008) (Carl Zeiss, Oberkochen, Germany). BS was estimated from the number of viruses present in phage-filled cells (i.e., burst state) as well as from the ratio between the maximum concentration of the free viruses measured after lysis over the maximum concentration of infected cells before cells lyse (Jacquet et al., 2013).

#### Pulse Field Gel Electrophoresis

Prior to pulse field gel electrophoresis (PFGE), 70 ml lysates were filtered through a 0.22-μm pore-size PVDF membrane and the viruses concentrated using a Centricon Plus-70 centrifugal filter (Millipore) according to the manufacturer’s instructions. Approximately 10^9^ viral particles were embedded in a PFGE plug following Zhong et al. (2014). Then the PFGE plugs were loaded into well of a 1% agarose gel (Invitrogen) together with the molecular weight markers (a lambda and a Mid-Range ladder; New England BioLabs). Electrophoresis was performed using a CHEF-DR II system (Biorad, Germany) with the following conditions: 14°C, 200 V, 2–10 s switch time for 22 h and 0.5× TBE buffer (90 mM Tris-borate and 1 mM EDTA, pH 8.0). Gels were stained with ethidium bromide for 45 min and washed with distilled water. The DNA bands were visualized using a Geldoc (BioRad).

#### Host Range Experiments

Host range was tested by adding 1 ml containing ∼10^9^ S-LBS1 particles to 4 ml in 24-well microplates of the following exponentially growing freshwater culture strains from the Thonon Culture Collection (TCC) and Pasteur Culture Collection (PCC). Infectivity was tested on 20 strains of PE-rich *Synechococcus* isolated from Lakes Annecy and Bourget, PC-rich *Synechococcus* (PCC 6301, 6311, 6707, 6715, 7917, 7918, 7941, 7952, 9004, and 9005), PC-rich *Synechocystis* (PCC 6308, 6803, 6905, and 7509), colonial (*Microcystis aeruginosa*, TCC 80) and filamentous cyanobacteria (*Planktothrix rubescens*, TCC 29), as well as eukaryotic microalgae belonging to the *Chlorophyceae* (*Chlorella sorokiniana*, TCC 211 and *Scenedesmus acutus*, TCC 141), and *Cryptophyceae* (*Cryptomonas* sp, TCC826). Controls received only sterile medium. All plates were grown under the same conditions as described above, and monitored daily.

#### Induction of Lysogenic Viruses of PE-Rich *Synechococcus*

Technical details to address the prevalence of lysogeny within the different TCC cultures of *Synechococcus* can be found in **Supplementary Methods**.

#### Viral Genomic DNA Extraction, Purification, and Sequencing

Approximately 60 ml of viral lysate was filtered twice through 0.2 μm pore-size PVDF membranes (GVWP, Millipore) using a clean and sterile Sterifil Filtering Unit (Millipore) connected to a vacuum pump. Viral particles were concentrated from the filtrate using ultracentrifugation at 119,577 × *g* for 5 h at 8°C (45Ti rotor Beckman Coulter, Chénard et al., 2015). The pellet was resuspended using 500 μl DNase and RNase free water (molecular quality water, Invitrogen). To remove any free DNA, 200 μl of concentrated viruses was treated using DNase I (Amplification grade, Invitrogen) at room temperature for 15 min. The enzyme was deactivated by adding EDTA (2.5 mM final) and incubating at 70°C for 10 min. Viral genomic DNA was extracted from the DNase I treated viral concentrate using PureLink^®^ Viral RNA/DNA Mini Kit (Invitrogen), and then sheared to between 200 and 500 bp fragments (mean ∼300 bp) using a Covaris M220 ultrasonicator (Covaris, Woburn, MA, United States) and purified using Agencourt AMPure XP beads (Beckham Coulter). The sequencing library was built based on 100 ng of this purified DNA using NxSeq^®^ AmpFREE Low DNA Library Kit (Lucigen, Middleton, WI, United States), and sequenced using Illumina HiSeq^®^ 2500 with 2 × 100 bp pair-end reads at the Biodiversity Research Centre at The University of British Columbia.

#### Genome Assembly, Gene Prediction and Annotation

Adapters were trimmed using Trimmomatic-0.30 (Bolger et al., 2014), quality checked with fastQC version 0.11.5 (Andrews, 2010), and assembled using SPAdes version 3.9.0 (Bankevich et al., 2012) with Kmer settings of 21, 33, 55, 77, and 99. To examine sequencing coverage of the contigs, the raw reads were aligned and mapped back to each contig using the Burrows-Wheeler Aligner (BWA, release 0.7.16) and Sequence Alignment/Map tools (SAMtools, release 1.8) (Li et al., 2009). Sequencing yielded approximately 13.6 million paired-end reads of 36–100 bp. In total, 2923 contigs were obtained, of which 585 were >1 kb, the largest of which was 34,641 bp in size. Approximately 66.4% of raw reads could be mapped back to the 34,641 bp viral contig with an average and homogeneous coverage on each base of ∼24,665. This viral contig, referred later as S-LBS1, was subjected to further analysis for gene prediction and annotation as follows.

Putative coding sequences (CDSs) in S-LBS1 were predicted using GeneMark version 2.5 (Lukashin and Borodovsky, 1998) and GLIMMER version 3.02 (Delcher et al., 2007). When the predictions differed, the longer of the two was kept. The predicted open reading frames (ORFs) were translated and assigned putative functions using BLASTp to compare them with protein sequences in the GenBank non-redundant (nr) database. Sequences with *e*-values <10^-4^ were considered to be homologs. Protein family and domain predictions in the sequences were conducted by searching against the Conserved Domain Database (Marchler-Bauer et al., 2013) and Pfam database (Finn et al., 2014) (*E*-value cut-off <0.0001). InterproScan 5 (Jones et al., 2014) was used to predict the transmembrane proteins and signal peptides against Phobius (Käll et al., 2007). The genome was also analyzed for regulatory elements and motifs such as tRNA genes, promoter motifs, and transcriptional terminators. tRNA genes were identified using tRNAscan-SE (Lowe and Eddy, 1997). Putative promoter motifs were identified using PePPER (de Jong et al., 2012). Rho-independent terminators were identified using ARNold (Lesnik et al., 2001). Phage termini and DNA packing strategy were predicted using PhageTerm (Garneau et al., 2017) with a mapping coverage setting of 20,000. S-LBS1 genome sequence was deposited in GenBank under accession number MG271909. Raw Illumina reads were deposited in NCBI SRA database under accession number SRP142506.

#### Comparative Genomic Analysis

To compare S-LBS1 and the other cyanosiphovirus genomes in GenBank (**Supplementary Table S1**), a pan genome analysis that identifies the core and accessory genes, was carried out using Roary version 3.11.2 (Page et al., 2015) applying standard parameters (minimum BLASTp identity of 50%). Given these cyanosiphovirus genomes have been annotated using different gene prediction programs, to ensure consistent gene calling, all genes within the genomes were re-annotated using Prokka (Seemann, 2014) prior to the analysis using Roary. In particular, the presence/absence of homologous genes between cyanosiphoviruses were determined (**Supplementary Data Sheet 1**). To cluster phages with similar gene contents, a matrix was built based on either the presence/absence of genes, or the percentage of genes shared between two phages (number of homologous genes shared between two phages/total genes of two phages), then after a heatmap was constructed and the dendogram used to cluster the phage genomes based on Bray–Curtis similarity.

#### Phylogenetic Analysis

The terminase large subunits of S-LBS1 were aligned using MAFFT version 7 (Katoh et al., 2002), with representatives for each defined cluster from other studies (**Supplementary Table S2**). Phylogenies using both the Bayesian-inference and maximum-likelihood methods were conducted based on the multiple alignments. Bayesian inference was performed using MrBayes 3.2.1 (Ronquist et al., 2012) with two runs, four chains, one million generations, sampling every 100 generations, a burn-in value of 25%, and mixed models of amino-acid substitution. The maximum likelihood phylogeny was constructed using RAxML (Stamatakis et al., 2008) with 100 bootstrap replicates and using the LG model and gamma-distributed substitution rates. For phylogenetic analysis of siphovirus-gp157 (a phage resistant protein—see the Section “Results”), homologs were obtained by blast (BLASTp, *E*-value = 0.01) against the NCBI nr database using acid-amino sequences from gp157 of S-LBS1 as the query. The phylogenetic analysis was conducted as described above.

#### Recruitments of Metagenomic Reads to S-LBS1 Genome

To explore the presence of S-LBS1-like viruses in aquatic environments, reads were recruited from viral metagenomic data (**Supplementary Table S3**) onto the genome of S-LBS1 as proposed by Zhao et al. (2013). Briefly, each metagenome (**Supplementary Table S3**) was firstly made into a BLAST nucleotide database and queried with the predicted proteins of S-LBS1 using tBLASTn with an *e*-value set at 10^-4^. Putative metagenomic nucleotide reads were then extracted from each metagenome and used as query to blast (BLASTx, *e*-value = 10^-4^, max_target_seqs = 1) against a viral protein database containing the predicted proteins of (i) S-LBS1, (ii) phages listed in **Supplementary Table S4**, and (iii) other 2036 phages genomes of NCBI Reference Sequence Database (RefSeq, released on 11 November 2017) that are not present in **Supplementary Table S4**. If the returned best hit of each metagenomic read to the viral protein database of phage genomes was related to S-LBS1 but other phages, this read was then kept and recruited for S-LBS1-like viruses. At last, the recruited reads were mapped on the S-LBS1 genome based on their percent amino-acid identity using ggplot2 (Wickham, 2009) in R. The total number of hits to S-LBS1 was normalized by dividing by the length of the S-LBS1 genome (in kb) and the size of the metagenome (number of reads recruited per kb of genome/size of the database in Gb), which provides a normalized measure to compare recruitments by different sized contigs versus several metagenomes. Similar recruitment analysis was also conducted for other phages and are listed in **Supplementary Table S4**.

## Results

### General Features of the Host *Synechococcus* (Strain TCC793)

The cyanophage S-LBS1 infected the picocyanobacterium *Synechococcus* strain (TCC793), isolated from Lake Bourget in March 2011. The cells were about 2.1 ± 0.5 μm in length by 0.9 ± 0.1 μm in width, with a cell volume of 1.2 μm^3^ and were pink to red in color at irradiances <100 μmol quanta m^-2^ s^-1^, and yellow at irradiances >300 μmol quanta m^-2^ s^-1^. Based on phylogenetic analysis of 16S rRNA gene sequences and flow cytometric analysis, TCC793 was PE-rich and a close relative of sub-alpine cluster I cyanobacteria (**Supplementary Figure S1**). The maximum growth rate found for this strain was 0.3 d^-1^. Fluorescence excitation spectra had a maxima at 525 and 560 nm, indicating a phycoerythrobilin-rich PE, devoid of phycourobilin (Six et al., 2007). Fluorescence emission spectra had PE maxima at 580 and 664 nm, related to the fluorescence emission of PC and the phycobilisome terminal acceptor (allophycocyanin and chlorophyll *a*). The *in vivo* absorption spectrum confirmed these observations, placing *Synechococcus* strain TCC793 as pigment type 2 (Cabello-Yeves et al., 2017). The three major lipophilic pigments eluted in the order of zeaxanthin, chl *a* and β-carotene (**Supplementary Figure S2**).

### General Features of S-LBS1

**FIGURE 1:**
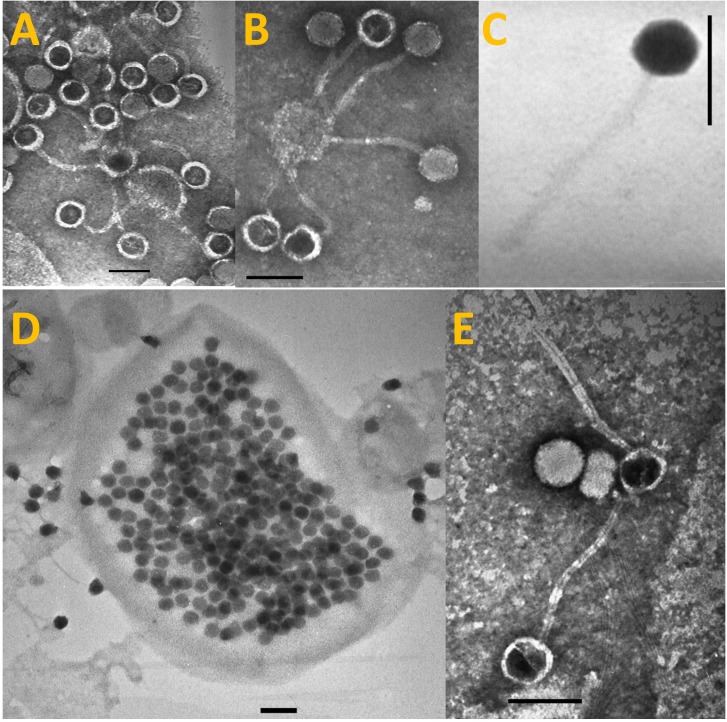
Transmission Electron Microscopy (TEM) images of *Synechococcus* phage S-LBS1, present as free particles outside of cells **(A–C,E)** or as immature progeny phage particles within the infected cell **(D)**. The scale bar is 100 nm.

Transmission electron microscopy revealed that S-LBS1 was a hexagonal particle about 75–80 nm in diameter with a flexible, non-contractile tail 185–190 nm in length (**Figure 1**), consistent with members of the family *Siphoviridae*. Infectivity was not chloroform sensitive, indicating the absence of lipids outside of the capsid (not shown). Infection experiments revealed that S-LBS1 required a relatively long period, of about 4 days, before cell lysis occurred (**Supplementary Figure S3**), and a BS of ∼400 (387 ± 42) viruses per cell (**Figure 1**). Host range studies with S-LBS1 was tested on a large number of isolates of cyanobacteria and eukaryotic algae listed in the Section “Materials and Methods”. Lysis only occurred with *Synechococcus* sp. TCC793, suggesting a narrow host range.

### Genomic Analysis of S-LBS1

The 34,641 bp viral contig was obtained after *de-novo* assembly, to which 66.4% of raw reads could be mapped back, with an average ∼24,665-fold coverage evenly spread over the contig (**Supplementary Figure S4**). This result is consistent with a band slightly larger than 33 kb on the PFGE gel (**Supplementary Figure S5**). It suggests that the genome of S-LBS1 is linear and dsDNA, because circular dsDNA, ssDNA, or RNA would otherwise remain trapped in the PFGE slot, migrate differently, or result in smearing (Alduina and Pisciotta, 2015). The genome termini was predicted to be at position 25,643 based on mapping Illumina raw reads back to the S-LBS1 genome using PhageTerm (Garneau et al., 2017). S-LBS1 seems employing a headful packing strategy similar to phage P, in which a concatemer containing several copies of the genome is firstly made prior to packing, then the terminase initiates packaging at a specific packaging site (pac site) and cuts this concatemer at various positions until the phage head is full (Casjens and Gilcrease, 2009; Garneau et al., 2017).

**FIGURE 2:**
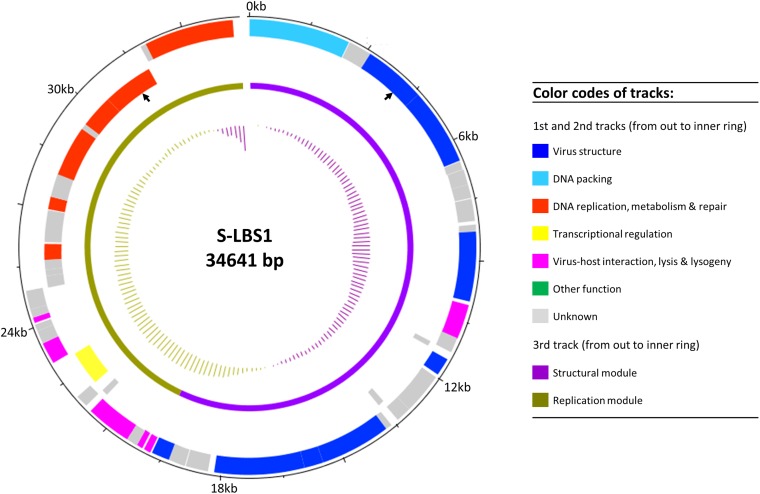
Genomic map of S-LBS1. Circles from outmost to innermost correspond to (i) predicted coding sequences on forward strand and (ii) reverse strand; (iii) defined genomic modules (structural and replication modules); and (iv) GC content plotted relative to the mean of G+C of genome at 60.2%. The black arrows show where the putative prophage attachment sites are located.

In total, 52 ORFs were predicted and annotated (**Figure 2** and **Supplementary Table S1**), although about half could not be assigned a putative function. The genome architecture of S-LBS1 was organized into two gene clusters. Module I (structural module; **Figure 2**) contained genes essential for virion structure, DNA packing, and lysogeny (e.g., integrase), while Module II (replication module) consisted of genes related to virus replication, virus–host interaction, DNA repair, DNA metabolism and lysis. The average GC content of the genome was 60.2%, although the structural module was characterized by a lower average GC ratio of 58.3%, with a particularly low value for the gene encoding the tail fiber (53.8%).

**FIGURE 3:**
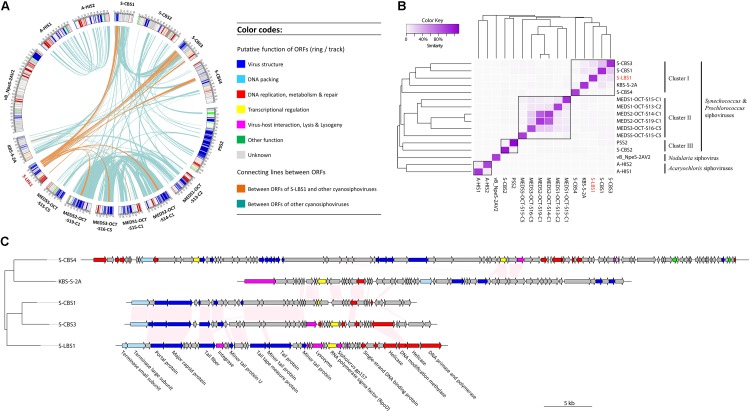
Schematic circos plot **(A)** displays line connections between homologous ORFs (BLASTp, acid amino identity >50%) in 16 cyanosiphoviruses (**Supplementary Table S1**). The lines are highlighted using orange for ORFs with homologs in S-LBS1, and light-blue if an ORF occurs in between two cyanosiphoviruses. Putative assigned functions of the ORFs are color-coded as indicated in the legend. To display similarity in gene content between two phages, a heatmap **(B)** was built on a matrix based on the percentage of genes shared between two phages (number of homologous genes shared between two phages/total genes of two phages). The dendogram on the left and on the top of the heatmap was clustered based on Bray–Curtis similarity. **(C)** Compares the genomes of five siphoviruses infecting *Synechococcus* sp. Pink panels connect homologous ORFs between two phages. The phylogenetic tree on the left was based on the alignment of DNA sequences of entire genomes using MAUVE (Darling et al., 2004). The color code is the same as in **(A)**.

Comparative analysis revealed 998 genes within 16 cyanosiphovirus genomes, yet core genes could not be detected (**Supplementary Figure S6**). Nevertheless, S-LBS1 shared homologous genes (**Figure 3A**), mostly in the structural module, with eight *Synechococcus* siphoviruses, four from Chesapeake Bay (S-CBS1, S-CBS3, S-CBS4, and KBS-S-2A) and four from the Mediterranean Sea (MEDS2-OCT-S19-C1, MEDS3-OCT-S16-C5, MEDS1-OCT-S15-C1, and MEDS2-OCT-S14-C1). The number of homologous genes with S-LBS1 was much higher for six out of the eight siphoviruses, with little overlap with KBS-S-2A and MEDS2-OCT-S19-C1. There was no overlap between S-LBS1 and the marine *Prochlorococcus* siphovirus PSS2 and three marine *Synechococcus* siphoviruses (S-CBS2, MEDS5-OCT-S13-C2, and MEDS5-OCT-S15-C5). The number and content of shared genes shows how closely the genomes and genomic regions are related, with 13 cyanosiphoviruses being interconnected to different extents (**Figure 3A**). However, none of the cyanophages shared similar genes with vB_NpeS-2AV2 (**Figure 3A**), a new lineage of cyanosiphovirus recently isolated from the Baltic Sea that infects the filamentous nitrogen-fixing cyanobacterium, *Nodularia* sp. (Coloma et al., 2017). Additionally, the siphoviruses A-HIS1 and A-HIS2 that infect the unicellular cyanobacterium *A. marina* (Chan et al., 2015), did not share homologous genes with siphoviruses infecting unicellular cyanobacteria in the genera *Synechococcus* or *Prochlorococcus* (**Figure 3A**). These results imply that siphoviruses infecting filamentous and unicellular cyanobacteria may form different genetic lineages.

Indeed, an analysis of the gene content of the 16 cyanosiphoviruses (**Figure 3B**) split the viruses based on their hosts into those infecting isolates of the genera *Synechococcus* and *Prochlorococcus*, *Nodularia* and *Acaryochloris*. Within the *Synechococcus* and *Prochlorococcus* siphoviruses, there were three clusters. Cluster I grouped the *Synechococcus* siphoviruses from Chesapeake Bay and Lake Bourget; Cluster II encompassed *Synechococcus* siphoviruses from the Mediterranean Sea; Cluster III grouped *Prochlorococcus* siphovirus PSS2 and *Synechococcus* siphovirus S-CBS2. These results show that there is distinct evolutionary lineages among cyanosiphoviruses. It is noteworthy that similar clustering result was also obtained when the presence/absence of these 998 genes was analyzed (**Supplementary Figure S6**).

**FIGURE 4:**
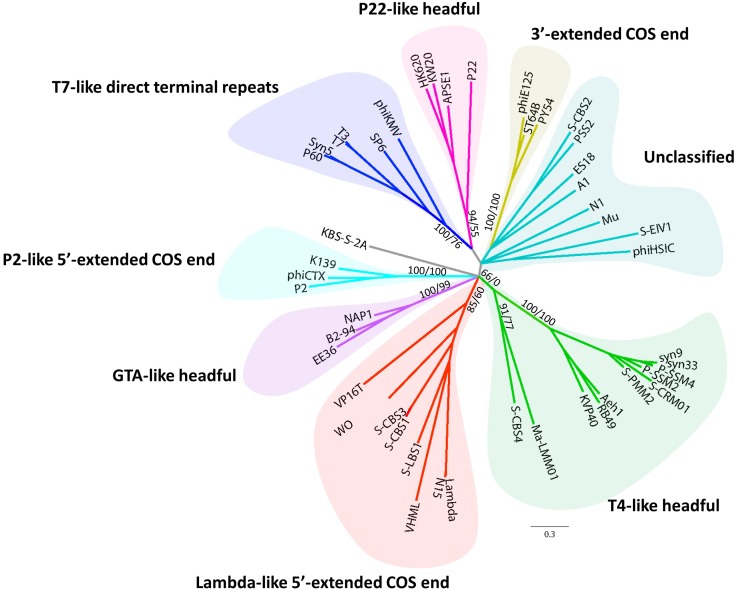
Unrooted Bayesian phylogenetic tree of 47 inferred amino-acid sequences of the terminase large subunit (*terL*), obtained from S-LBS1 and others phage representatives (**Supplementary Table S2**). Values shown at the nodes of the main branches are the Bayesian inference (BI) clade credibility and Maximum Likelihood (ML) bootstrap values, and are reported as BI/ML.

Phylogenomic analysis (**Figure 3C**) of cyanosiphovirus genomes from Cluster I confirmed that S-LBS1 was closely related to S-CBS3 and S-CBS1, two *Synechococcus* siphoviruses isolated from Chesapeake Bay. The three phages had similar genome sizes between 30 and 35 kb, and shared a similar genome architecture with a structural model comprising genes for virion structure and DNA packaging, and a replication module with genes for DNA replication and repair, metabolism, transcriptional regulation, and virus–host interaction (**Figure 3C**). S-LBS1 shared the most homologous ORFs with S-CBS3 (11 ORFs) and S-CBS1 (8 ORFs). High similarity was found in the structural proteins (i.e., major capsid protein, portal protein, tail fiber, minor tail). Phylogenetic analysis showed that the large terminase subunit (*terL*) of S-LBS1, S-CBS3, and S-CBS1 was closely related to those lambda-like phages (**Figure 4**). Despite the high degree of mosaicism in phages (Hendrix et al., 1999), phylogenomic, phylogenetic and gene content analyses, reveals closer evolutionary relationship among S-LBS1, S-CBS3, and S-CBS1 compared to other cyanosiphoviruses (**Figures 3A**, **4** and **Supplementary Figure S6**), and despite the very different environments from where they were isolated.

Despite the above similarity in genome size, genome architecture, and evolutionary history of *terL* between S-CBS1, S-CBS3, and S-LBS1, there are differences in gene content and sequences. First, S-LBS1 have genes that S-CBS1 and S-CBS3 do not have, such as integrase, terminase small subunit (*terS*), siphovirus-gp157, and DNA primase-polymerase (**Figure 3C**). DNA methylase and single-strand DNA binding protein (*ssb*) occur in S-LBS1 and S-CBS3, but are absent in S-CBS1. Second, S-LBS1 lacks genes for exonuclease, endonuclease and endodeoxyribonuclease, which are found in S-CBS1 and S-CBS3. Third, despite coding the same gene among genomes, the amino-acid sequences can be divergent, as is the case for RNA polymerase sigma factor *rpoD*, lysozyme, and tape-measure protein; the amino-acid identity between S-LBS1 and S-CBS1 or S-CBS3 for these sequences is less than 50% and can be as low as ∼35%. Fourth, only S-LBS1 has two copies of helicase, both of which belong to the DEAD/H helicase superfamily (**Supplementary Table S5**). One helicase (gene_A047) harbors a *res* subunit of Type III restriction enzyme (pfam04851) and is likely a helicase-related protein involved in DNA repair; the other is located upstream of DNA polymerase, and does not contain this putative restriction enzyme, and is likely involved in DNA replication. Overall, S-LBS1 is closer to S-CBS3 in terms of genome architecture and gene content (**Figure 3C**).

### S-LBS1-Like Viruses Are Widespread in Aquatic Environments

**FIGURE 5:**
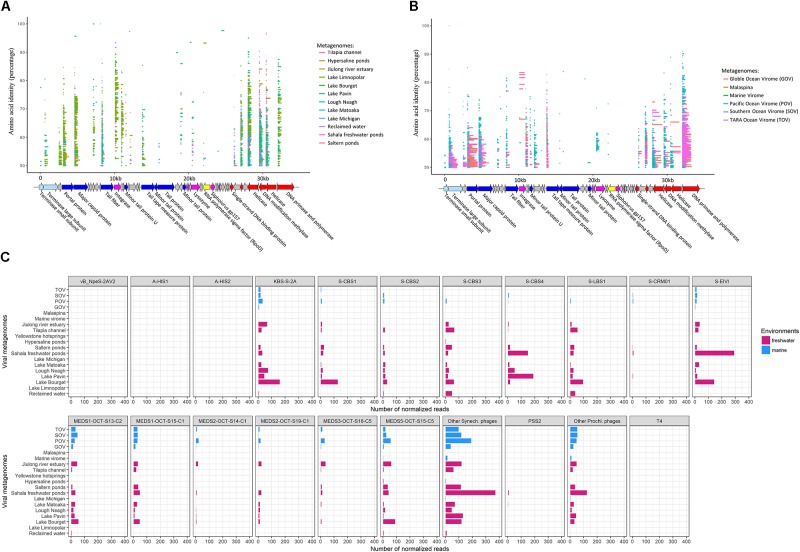
Prevalence of S-LBS1-like sequences in environmental viral metagenomic data. Fragment recruitment of reads from environmental viral metagenomic data (**Supplementary Table S3**) onto the genome of S-LBS1. Each horizontal line represents a read recruited from publicly available **(A)** Freshwater viral metagenomic datasets, or **(B)** marine viral metagenomic datasets. The length of each line represents the BLAST query coverage (percent of the query sequence that overlaps the subject sequence) and the *y*-axis position of each line indicates amino-acid identity (percent similarity) of the metagenomic reads to the S-LBS1 sequences over the length of the coverage area. The color codes for ORFs are as shown in **Figure 3**. The normalized total number of recruited reads for S-LBS1 (amino-acid identity >50%) and other phages across environments are shown in **(C)**, respectively. The total number of hits to phage was normalized by dividing by the length of the phage genome (in kb) and the size of the database (number of reads recruited per kb of genome/size of the database in Gb), which provides a normalized measure to compare recruitments by different size contigs versus several metagenomic datasets.

To explore the potential occurrence of S-LBS1-like viruses in aquatic environments, translated reads from freshwater and marine viral metagenomic datasets were recruited to the translated S-LBS1 genome. There were 35 ORFs, including 19 with a putative function, that recruited reads (**Figure 5A**, amino-acid identity >50%) from 13 freshwater viral metagenomes. S-LBS1-like sequences were detected in all freshwater metagenomic datasets except the Yellowstone hotsprings, although the relative abundance recruited to each ORF varied among environments (**Figures 5A,C**). These freshwater environments represented a variety of ecosystems across a wide geographic range (**Supplementary Table S3**), implying a wide distribution of S-LBS1-like viruses in freshwaters. The highest proportion of reads with the greatest coverage of the S-LBS1 genome (i.e., 24 ORFs, **Figure 5A**) were recruited from Lake Bourget (**Figure 5C**), which is consistent with the isolation of S-LBS1 from this lake and biogeographic patterns observed in cyanophages (Marston et al., 2013; Hanson et al., 2016). Reads from Lake Bourget that recruited to the S-LBS1 genome range widely in similarity, between 50 and 100%, and largely recruited to ORFs related to structure, or DNA replication, repair, and metabolism (**Figure 5A**). Divergent sequences were detected for the portal protein, major capsid protein, integrase, helicase, and primase-polymerase, suggesting a high diversity of viruses similar to S-LBS1. Unlike the results for Lake Bourget, the terminase large subunit (*ter*L), tail fiber and lysozyme were divergent in metagenomic data from Lough Neagh and the Jiulong River estuary (**Figure 5A**), suggesting that distinct lineages of S-LBS1-like viruses might be present in these environments. Relatively few reads were recruited from metagenomic data from hypersaline ponds, Lake Limnopolar, Lake Michigan and Lake Matoaka (**Figure 5C**); thus S-LBS1-like viruses may not occur in these environments, or exist as prophage.

Six marine metagenomic datasets contained sequences with similarity to 32 ORFs in the S-LBS1 genome, including 22 with a putative function (**Figure 5B**). The detection of sequences with similarity to S-LBS1 in marine environments is not surprising given its genetic similarity to the marine isolates S-CBS1 and S-CBS3. Interestingly, the overall coverage of S-LBS1 ORFs by metagenomic reads is higher for marine than freshwater metagenomic data. Indeed, many S-LBS1-ORFs, including the portal protein, terminase large subunit, primase-polymerase, methylase, helicase and the major capsid protein are fully mapped by metagenomic reads from GOS and TOV; this could be explained by the higher sequencing depth in these databases.

Similar to some marine *Synechococcus* siphoviruses (e.g., S-CBS1, S-CBS2, S-CBS3, S-CBS4; Huang et al., 2012), S-LBS1-like viruses appear to be widespread in the oceans (**Figure 5B**). However, the number of normalized reads was lower compared to *Synechococcus* siphoviruses from the Mediterranean Sea (**Figure 5C**) and to both marine cyanomyoviruses and cyanopodoviruses, and also, it was lower to their occurrence in some freshwaters. This is consistent with the freshwater nature of S-LBS1. S-LBS1-like viruses display a similar distribution pattern across metagenomes to S-CBS3-like viruses, while it contrasts to S-CBS1-like viruses that overweighted in Lake Bourget and underweighted in Tilapia Channel and Reclaimed water. This indicates the presence of another population of cyanosiphoviruses in Lake Bourget. Likewise, it could also be true for the KBS-S-2A, MEDS5-OCT-S15-C5, and S-EIVL-like viruses since they were detected abundantly in the metagenome of Lake Bourget (**Figure 5C**; Chénard et al., 2015). In addition to Lake Bourget, cyanosiphoviruses seem also enriched in freshwater metagenomes of Lough Neagh, Lake Pavin and Sarala freshwater ponds. However, we did not detect a specific cyanosiphovirus dominance in these extreme environments (e.g., Yellowstone hotsprings, hypersaline ponds) compared to other terrestrial freshwater environments.

## Discussion

### S-LBS1 Has a Large BS

S-LBS1 produces approximately 400 viral particles per infected cell, which is a higher BS compared to other known cyanophages to date. BS for phages infecting isolates of *Synechococcus* range widely. For cyanosiphoviruses, BSs have been reported of 250 for S-BBS1 (Suttle and Chan, 1993), 200 for S-CBS1 and S-CBS3 (Huang et al., 2012), and 60 for S-CBS2 and S-CBS4 (Huang et al., 2012), all considerably less than reported here. For cyanomyoviruses, BSs as low as 22 have been reported for S-PM2 (Wilson et al., 1996), and have ranged between 93 and 324 for other isolates (Suttle and Chan, 1994).

### S-LBS1 Has Lysogeny Potential

Lysogeny, where the phage genome integrates into host chromosome to form a prophage, appears to be rare in unicellular cyanobacteria. Integrase (a.k.a. site-specific recombinase) is essential and allows site-specific integration of the phage chromosome into the host via integrative recombination between the attP and attB sites of phage and host, respectively. This yields a prophage flanked by two prophage att sites, attL and attR (Landy and Ross, 1977). The prophage can excise the genome and enter the lytic cycle when triggered by environmental factors (Weinbauer, 2004). S-LBS1 encodes a putative integrase (gene_A12, pfam00589) belonging to the lambda-like tyrosine recombinase family, and two putative prophage attachment sites (motif sequence: CAGCAGCCGCTTGG); one is located between sites 4147 and 4160 within an ORF encoding the phage portal protein, and the other is between sites 31,346 and 31,359, within an ORF encoding helicase (**Figure 2**). This is the first report of an integrase in a siphovirus infecting *Synechococcus*. This suggests that S-LBS1 can lysogenize its *Synechococcus* host. Likewise, another integrase-containing cyanosiphovirus, PSS2, that infects a strain of *Prochlorococcus*, was also reported but it was obtained from Atlantic seawater (Sullivan et al., 2009). Also, S-LBS1 is predicted to be a temperate phage (average probability = 55.7%) using Phage Classification Tool Set (PHACTS^1^) based on its genome sequence. Despite the genetic potential for lysogeny, it is noteworthy that we did not find evidence of integration of S-LBS1 into the genome of *Synechococcus* sp. TCC793, based on induction using mitomycin C. More work needs to be completed to confirm S-LBS1 is a prophage.

Evidence for lysogeny in unicellular cyanobacteria has been observed in marine (McDaniel et al., 2002; Ortmann et al., 2002; McDaniel and Paul, 2005) and fresh (Dillon and Parry, 2008) waters, by induction in natural cyanobacterial populations using mitomycin C. Despite the genetic potential for lysogeny (e.g., PSS2, S-LBS1), inducible prophage have not been found in isolates of *Synechococcus* or *Prochlorococcus*, although prophage sequences have been found in unicellular freshwater cyanobacteria (Huang et al., 2012). Indeed, no prophage was found in genomes of 143 marine *Prochlorococcus* and 25 *Synechococcus* in CyanoBase^2^, although numerous attB sites were detected, particularly in *Prochlorococcus*. However, two intact prophages were identified from freshwater *Synechococcus* genomes using PHAST^3^. One was a 46.8 kb prophage in the genome of *Synechococcus elongatus* PCC 7942 (Accession#: CP000100; region: 711254-759932), and the other, a 42.2 kb prophage in the genome of *Synechococcus* sp. PCC 6312 (Accession#: CP003558; regions: 1104970-1147248). The former appears to be a siphovirus since its terminase large subunit and integrase are similar to those found in S-CBS3 and PSS2, respectively. With a transposase in its genome, the later appears to be a Mu-like myovirus. These findings suggest that prophages occur in *Synechococcus* and *Prochlorococcus*. This is consistent with observations that diverse and divergent S-LBS1-like integrases are relatively common in freshwater viral metagenomic data, while few are found in marine datasets (**Figure 5**). Given that marine phages have the genetic potential for lysogeny (e.g., PSS2), and infectious cyanophage can be induced from natural populations of *Synechococcus* using mitomycin C (Ortmann et al., 2002), the absence of prophage sequences in marine *Synechococcus and Prochlorococcus* isolates suggests that these cyanobacteria may be able to cure prophage from their genomes, or that prophage are induced when cells are brought into culture.

### Possible Resistance Mechanisms Against S-LBS1 in *Synechococcus*

**FIGURE 6:**
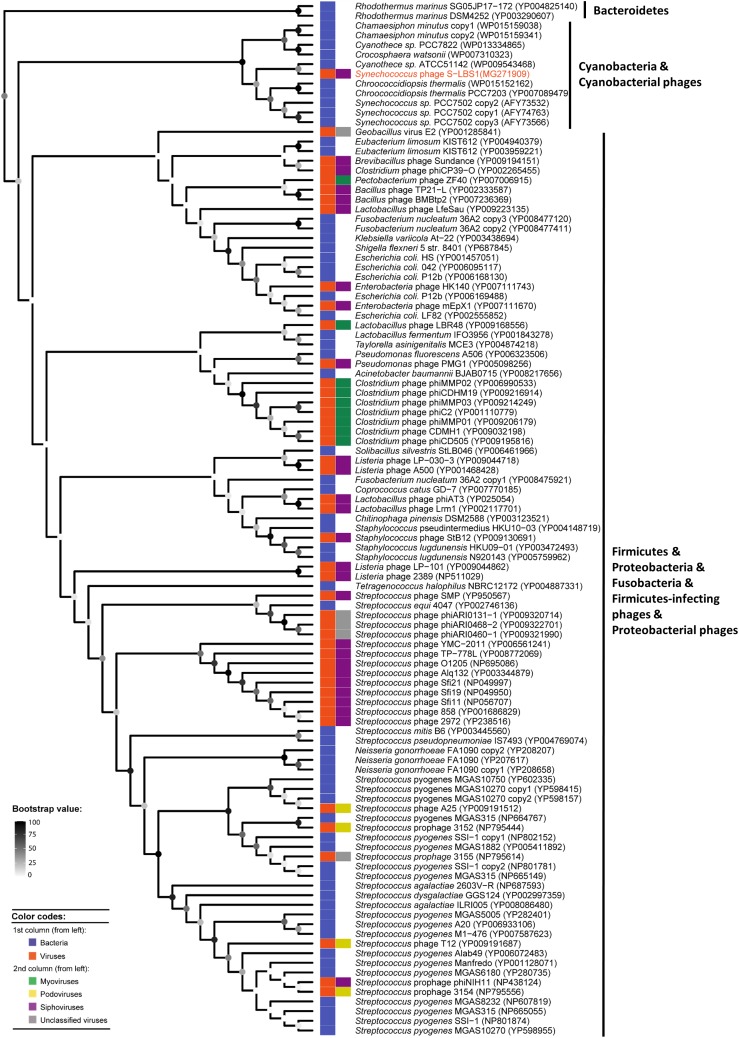
RaxML phylogenetic tree of 107 inferred amino-acid sequences similar to Siphovirus-gp157 (pfam05565), obtained by blast against the NCBI nr reference protein database using Siphovirus-gp157 from S-LBS1 as the query. Points at the nodes show maximum-likelihood (ML) bootstrap values ranging from 0 (white) to 100 (black). Phylogenetic tree leaves were labeled by the name of the organism that contains this shiphovirus-gp157 sequence, followed by the NCBI accession number in parentheses. Phylogenetic tree leaves were also annotated by two columns of color strips; the first column distinguish the bacterial and viral shiphovirus-gp157 sequences; the second column differs the morphological types of viruses (myovirus, podovirus, siphovirus, and unclassified) in which the shiphovirus-gp157 sequences were found.

During infection experiments, *Synechococcus* recovered about 2–3 weeks after infection while the virus titer was still high (**Supplementary Figure S7**). After re-infection with a new viral lysate, there was no decline in host cells or phage concentrations. This may indicate phage-resistant *Synechococcus* cells in the lysed cultures (Lennon et al., 2007). Based on the genetic potential, two resistance mechanisms against S-LBS1 can be hypothesized. Firstly, the resistant phenotype might be due to the lysogeny potential of S-LBS1. For example, in the Arbitrium system in the *Bacillus* siphophage of the SPbeta group, ancestral phages employ signal peptides to communicate with their host to decide between lytic and lysogenic lifestyles in offspring phages (Erez et al., 2017). This feedback mechanism from the phage to the host would lead to transition between lytic and lysogenic life cycles, and therefore, resistance against phage infection. We identified three putative signal peptides in S-LBS3 (**Supplementary Table S5**). Whether this phage is a signal dependent process needs to be examined. Secondly, resistance might be related to siphovirus-gp157 (gene_A34), a resistance-related protein-encoding gene in S-LBS1 located downstream of the RNA polymerase sigma factor RpoD (**Supplementary Table S5** and **Figure 3C**). Siphovirus-gp157 (pfam05565) is a protein family constituent of viral and bacterial proteins related to the gp157 protein of *Streptococcus thermophilus* SFi bacteriophages. Bacteria possessing the gene encoding this protein have an increased resistance to bacteriophages (Foley et al., 1998). During infection by S-LBS1, siphovirus-gp157 would confer immunity to *Synechococcus*, but the molecular basis for resistance remains to be elucidated. To explore its diversity, the sequence of siphovirus-gp157 was searched against the NCBI nr database (BLASTp). There were 107 sequences that were similar to siphovirus-gp157 in genomes of bacterial phyla from Firmicutes, Bacteroidetes, Proteobacteria, Fusobacteria, and Cyanobacteria, as well as in phages infecting members of Firmicutes and Proteobacteria (**Figure 6**). They belong to the myo-, podo-, sipho-morphological types of dsDNA viruses, as well as, tail-less phages (**Figure 6**). This suggests that siphovirus-gp157 and its related resistance mechanism is not endemic to a specific viral morphotype. It is interesting to note that the majority of these siphovirus-gp157-containing organisms are not from aquatic environments. Indeed, no siphovirus-gp157-like sequences were found in either freshwater or marine viral metagenomic data (*e*-value cut-off: 0.0001). Even applying a less strict *e*-value cut-off of 0.1, the number of sequences were few. These aquatic siphovirus-gp157 homologs belonged to *Rhodothermus marinus* of the phylum Bacteroidetes, cyanobacteria and its phages (**Figure 6**). They were clustered together with strong bootstrap support values and were divergent from other siphovirus-gp157-containing bacteria and phages (**Figure 6**). This suggests that the acquisition of siphovirus-gp157 in aquatic Bacteroidetes, Cyanobacteria and cyanophages may be related to a recent recombination event. Whether the siphovirus-gp157 is derived from host cells or originates in viruses is unknown; hence, its ecological relevance remains to be studied.

### Absence of the Core Photosystem Genes psbA and psbD in Cyanosiphoviruses

The D1 protein of the core photosystem reaction center for PS-II turns over quickly and can be damaged by high light and UV; yet, many cyanophages of unicellular cyanobacteria require active photosynthesis for infection (Suttle, 2000; Bailey et al., 2004). Thus, about 2/3 of cyanomyovirus and cyanopodovirus isolates have the host-derived genes *psbA* and *psbD* that encode the core photosystem proteins D1 and D2, respectively (Wang and Chen, 2008; Sullivan et al., 2009; Huang et al., 2012; Dekel-Bird et al., 2013; Zhong and Jacquet, 2013). These auxiliary metabolic genes (AMGs) are expressed during infection, providing stable, sufficient and continuous energy for phage production (Lindell et al., 2005; Sharon et al., 2007). However, neither S-LBS1 nor 15 other sequenced cyanosiphoviruses contain *psbA* or *psbD*. We speculate that this absence may be explained by (i) the long latent period for siphoviruses that require slower or un-continuous energy supply, compared to viruses taking only a few hours to produce progeny during an acute lytic infection, (ii) the lysogenic nature of cyanosiphoviruses (Sullivan et al., 2009; Huang et al., 2012) because a lifestyle without any phage production could be an advantageous option for the host in case of energy and nutrients stress or shortage, or (iii) the production of light-stable D1 proteins by the cyanobacterial host (i.e., most freshwater *Synechococcus* possess the light-stable D1 proteins; Sicora et al., 2006) so that the replacement of unstable host D1 protein by stable viral D1 protein is therefore unnecessary. Hence, we propose today that cyanosiphovirus genomes are likely to be exempted of viral *psbA/D* and that other AMGs may replace them. Typically, the high-light-inducible-protein-encoding gene (*hli*), supposed to protect the photosynthetic apparatus from photodamage, is present in S-CBS2 (Huang et al., 2012).

### High Variability in Genomes of Siphoviruses Infecting Cyanobacteria

Our comparative analysis showed that core genes could not be detected in genomes of 16 sequenced cyanosiphoviruses, implying high degree of genetic variability in siphoviruses infecting cyanobacteria. Interestingly, the gene content analysis either based on the number of shared genes between genomes or the presence/absence of 998 genes within the 16 cyanosiphoviruses, could discriminate/distinguish cyanosiphoviruses based on their hosts, like those infecting genera *Synechococcus, Prochlorococcus*, *Nodularia* and *Acaryochloris* (**Figure 3B** and **Supplementary Figure S6**). This finding leads us to speculate that such genetic variability of cyanosiphoviruses may be the result of adaptive co-evolution between virus and host. Nevertheless, the core genes were also absent among all the 13 cyanosiphoviruses infecting *Synechococcus* and *Prochlorococcus*. This contrasts to cyanomyoviruses (Millard et al., 2009; Sullivan et al., 2010) and cyanopodoviruses (Huang et al., 2015) isolated from *Synechococcus* and *Prochlorococcus*, in which the core genes could be detected. It suggests that cyanosiphovirus genomes of unicellular cyanobacteria are highly divergent compared to those of cyanomyoviruses and cyanopodoviruses. However, it is noteworthy that nine core genes could be detected within these three closely related *Synechococcus* siphoviruses (S-LBS1, S-CBS1, and S-CBS3), although they have been isolated from freshwater lakes and marine estuarine, respectively. This is consistent with the detection of cores genes for cyanomyoviruses (Millard et al., 2009; Sullivan et al., 2010) and cyanopodoviruses (Huang et al., 2015) between freshwater and marine environments. All together, these results highlight that host-selection seems overweighting the environment-selection in driving the diversification of cyanosiphoviruses. Differently said, virus–host interaction shall be the main driving force for the diversification of cyanosiphoviruses.

## Conclusion

This work reports the first genome and genomic analysis of a freshwater cyanosiphovirus. With the largest BS and the longest period needed after infection before cells lyse ever found in a cyanosiphovirus infecting a PE-rich picocyanobacterium, atypical and original genomic features as well as a widespread distribution in aquatic systems, S-LBS1 and related viruses are likely to be key components in controlling and structuring *Synechococcus* populations. This report therefore fills a gap in the database where cyanosiphovirus genomes from freshwaters are still absent to date. Certainly, this will lend a support to those who want to investigate cyanosiphoviruses in freshwater environments, using either PCR- or metagenomic-based methods.

## Author Contributions

KXZ wrote the article and contributed to the genomic analysis. CAS helped with writing the article. A-CB helped with writing the article. ED made the PFGE analysis. JoC made the TEM analysis. AC and JeC contributed to the genomic analysis. CS made the HPLC and pigment analysis. SJ isolated the virus and the host, made the FCM analysis, and wrote the article.

## Conflict of Interest Statement

The authors declare that the research was conducted in the absence of any commercial or financial relationships that could be construed as a potential conflict of interest.

## Footnotes

1 http://edwards.sdsu.edu/PHACTS/index.php

2 http://genome.microbedb.jp/cyanobase

3 http://phast.wishartlab.com/
