# Supplementary material for: A New Freshwater Cyanosiphovirus Harboring Integrase
Source: Front Microbiol. 2018 Sep 19;9:2204. doi: 10.3389/fmicb.2018.02204 (PMC6157547; doi:10.3389/fmicb.2018.02204)
Supplement: Supplementary file 2 [file Data_Sheet_2.docx]

***Supplementary Material***

**A new freshwater cyanosiphovirus harbouring integrase**

Kevin Xu Zhong^1,2^, Curtis A. Suttle^2,3,4^, Anne-Claire Baudoux^5^, Evelyne Derelle^6^, Jonathan Colombet^7^, Anna Cho^3^, Jessica Caleta^3^, Christophe Six^5^,

Stéphan Jacquet^1^*

1. INRA, UMR 042 CARRTEL, 75 bis Avenue de Corzent, 74203 Thonon-les-Bains cx, France
2. Department of Earth, Ocean, and Atmospheric Sciences, University of British Columbia, Vancouver, BC, Canada
3. Department of Microbiology and Immunology, University of British Columbia, Vancouver, BC, Canada
4. Department of Botany, and Institute for Oceans and Fisheries, University of British Columbia, Vancouver, BC, Canada
5. Sorbonne Universités UPMC Paris 06, CNRS, UMR7144 Adaptation et Diversité en Milieu Marin, Station Biologique de Roscoff, France
6. Integrative Marine Biology Laboratory (BIOM), CNRS UMR7232, Sorbonne Universities, Banyuls-sur-Mer, France
7. CNRS, Université Blaise Pascal, UMR 6023, Lab. Microorganismes, 24 Avenue des Landais, 63171 Aubière cx, France

*For correspondence.

Email: [stephan.jacquet@inra.fr](mailto:stephan.jacquet@inra.fr); Tel.  (+33) 4 50 26 78 12; Fax (+33) 4 50 26 07 60

**1. Supplementary Methods**

***Synechococcus isolation and characterization***

*Isolation* - In 2011*, Synechococcus* spp. were isolated, during the year from the 10 m depth of Lake Bourget at the reference station in the middle and deepest part of the lake, using the sorting function of a FACSCalibur flow cytometer (FCM). The phycoerythrin-rich picocyanobacteria were easily identified, discriminated, counted and sorted based on their scatter, phycoerythrin and chlorophyll fluorescence parameters (Personnic *et al.,* 2009; Cellamare *et al.,* 2010). Each *Synechococcus*-like cluster was sorted using the “exclusion mode” of the FCM sorting function, collected in a 50-ml falcon tube, and immediately transferred to a mix of BG-11 and Z liquid medium (50% : 50% by volume, respectively) to allow non-limiting growth conditions. After a few weeks under a 14 h:10 h Light:Dark cycle at 40-50 µmol quanta m^-2^ s^-1^ cool white light at 18°C, each *Synechococcus*-like group was purified by transferring to solid medium in Petri dishes using Z or BG11 medium mixed with 1% agarose for the plate and 0.8% agarose to spread the liquid culture of cyanobacteria. Colonies were then selected and transferred back to liquid Z medium. After several transfers between liquid and solid medium, each purified *Synechococcus*-like culture strain was maintained in liquid Z medium.

*Cell-size measurements* - Each culture was observed with an inverted microscope (Axiovert 135, Zeiss) using 1600-fold magnification. Images were obtained using a camera (Axiocam, Zeiss) and the size of at least 100 cells from each culture were measured using Axiovision software (Zeiss).

*Spectrofluorescence measurements* - Fluorescence spectra were recorded *in vivo* using a modified method of Six *et al.,* (2004). Briefly, exponentially growing cells were incubated for several minutes in the dark before *in vivo* excitation (emission at 680 nm) and emission (excitation at 530 nm) fluorescence spectra were recorded at room temperature in a quartz cuvette using a LS50B spectrofluorimeter (Perkin Elmer, USA) equipped with a red sensitive photomultiplier. Spectra were recorded at 150 nm min^-1^, monitored with the Perkin Elmer FL WinLab software and the slits were fixed at 8.0 nm. The absorption spectrum was recorded using a *mc²* spectrophotometer (SAFAS, Monaco).

*High Pressure Liquid Chromatography (HPLC) analyses -* To perform HPLC analyses, culture aliquots were washed and centrifuged at 20000 *g* and stored at -20°C until analysis. The cell pellets were extracted in 1 mL of 100% methanol and centrifuged twice at 20000 *g* to remove cell debris. All samples were prepared under subdued light at 4°C. The pigment composition was determined using a Hewlett-Packard HPLC 1100 Series System, equipped with a quaternary pump and diode array detector, and a Waters Symmetry C_8_ column of 150 x 4.6 mm, 3.5 μm particle size, based on a protocol adapted from Zapata *et al.,* (2000). Mobile phases were: A = methanol:acetonitrile:aqueous pyridine solution (0.025 M pyridine ; 45:35:20 v/v/v), and B = acetonitrile:methanol:acetone (60:20:20 v/v/v). The applied solvent gradient with a flow rate fixed at 1 mL min^-1^ was (time, % B): 0 min, 0 %; 5 min, 4 %; 18 min, 30%; 21 min, 30%; 26 min, 95 %; 36 min, 95 %; 38 min, 0 %. Chl *a* and carotenoids were detected by absorbance at 440 nm and identified by diode array spectroscopy and by their retention time (wavelength range: 350-750 nm; 1 nm spectral resolution).

**Virus characterization**

*Induction of lysogenic viruses of PE-rich Synechococcus -* We addressed the prevalence of lysogeny within the different TCC cultures of *Synechococcus* following Dillon and Parry (2008). Briefly, a 1 mg/L stock solution of mytomycin C (Sigma) was prepared in 0.02-µm filtered milliQ water and stored in the dark at 4°C. Ten ml aliquots of exponentially growing cultures from each culture were incubated with mitomycin C at final concentrations of 0 (control containing only water), 1, 5, and 20 µg mL^-1^ under a 14:10 L:D cycle at 40-50 µmol quanta m^-2^ s^-1^ PAR at 18°C for 2 weeks. Viruses were counted by FCM in one mL sub-samples taken at 0, 1, 7 and 14 d. Cyanophages were distinguished from bacteriophages based on both their signature and abundances by compared to the control sample in which phages were absent.

**2. Supplementary Figures and Tables**

**2.1. Supplementary Tables**

**Table S1** Cyanosiphoviruses used in comparative genomics analysis

**Table S2** The terminase large subunit (*terL*) sequences used for phylogenetic analysis.

**Table S3** Sources of viral metagenomic data used for fragment recruitment.

**Table S4** Phages used for fragment recruitment. It consists of (i) the currently 16 sequenced cyanosiphoviruses (included S-LBS1), (ii) freshwater *Synechococcus* phage S-CRM01 and S-EIVl, (iii) other *Synechococcus* phages (*Myoviridae* and *Podoviridae*, all from marine environments) from NCBI Reference Sequence Database (RefSeq, released on 11 November 2017), (iv) other *Prochlorococcus* phages (*Myoviridae* and *Podoviridae*, all from marine environments) from NCBI Reference Sequence Database (RefSeq, released on 11 November 2017), and (v) *Enterobacteria* phage T4 as the negative control.

**Table S5** The predicted ORFs of S-LBS1.

**2.2. Supplementary Figures**

**Figure S1** PhyML phylogenetic tree of 27 16S *rRNA* gene sequences obtained from *Synechococcus* *sp.* TCC793 and others *Synechococcus* representatives (Callieri *et al.,* 2013). Values shown at the nodes of the main branches are Maximum Likelihood (ML) bootstrap values. Phylogenetic tree leaves were labeled by the name of the organism, followed by the NCBI accession number in parentheses. *Synechococcus elongatus* PCC6301 was set as the root.

**Figure S2** Optical properties and pigment analysis of *Synechococcus* *sp.* TCC793. **A**: *in vivo* fluorescence excitation (emission at 680 nm) and emission (excitation at 530 nm) spectra; **B**: *in vivo* absorbance spectrum; **C**: High Pressure Liquid Chromatography chromatogram recorded at 440 nm. PEB: phycoerythrobilin, PCB: phycocyanobilin, PE: phycoerythrin, PC: phycocyanin, TA: phycobilisome terminal acceptor, Chl: chlorophyll, β-car: β-carotene.

**Figure S3** *Synechococcus sp*. TCC793 growing alone (filled symbol) and when infected by S-LBS1 (open symbol) under different MOI (multiple of infection). The cytogram of SYBR-green-strained VLP (virus-like particles) and *Synechococcus* that are not infected (**a**) and infected by S-LBS1 (**b**) were also included.


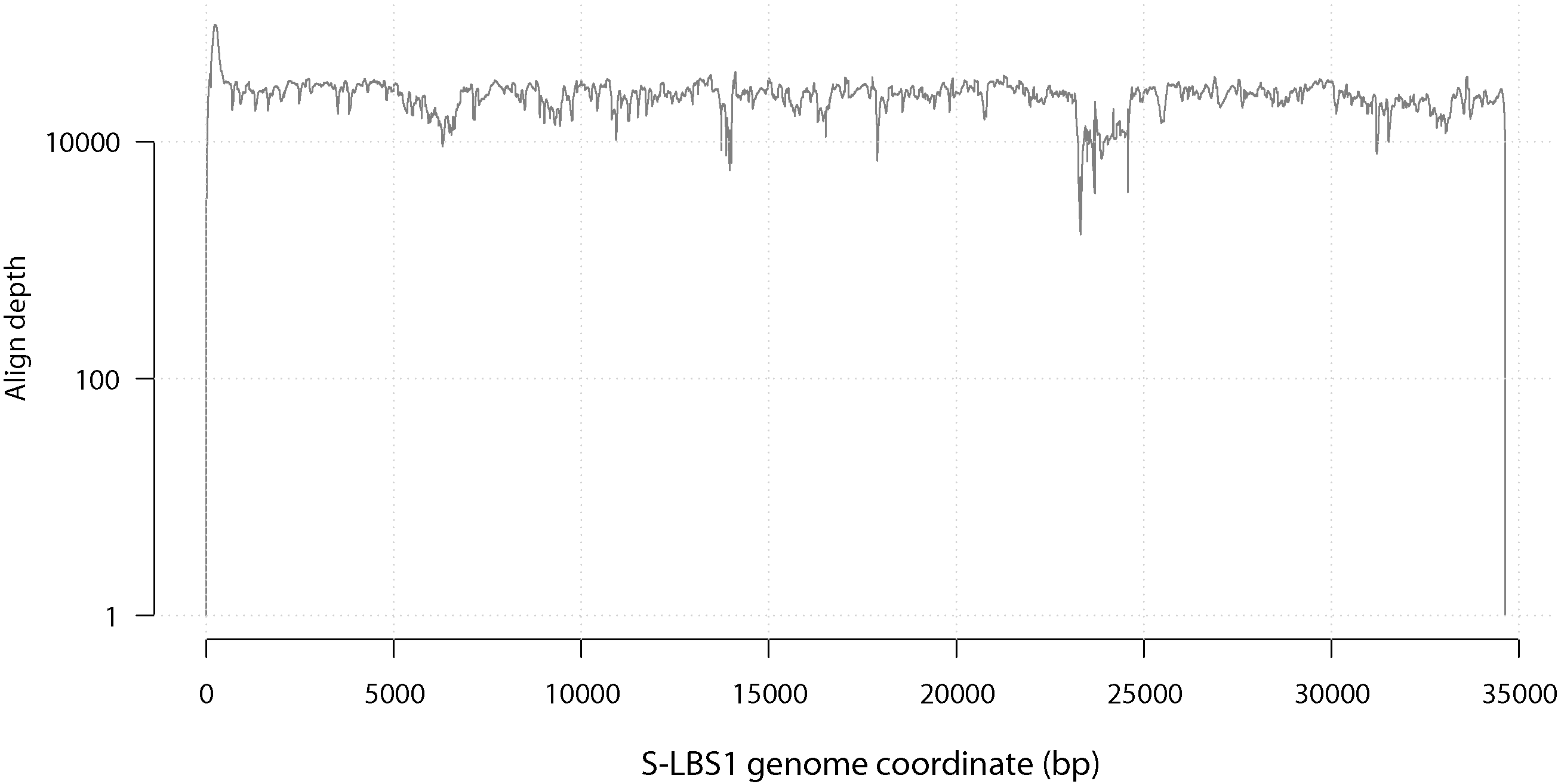


**Figure S4** Schematic plot showing the sequencing coverage of S-LBS1 when the raw illumina HiSeq 2x100bp PE reads were mapped back to genome of S-LBS1.

**Figure S5** Pulsed field gel electrophoresis (PFGE) showing the genome size of S-LBS1.

**Figure S6** Heatmap to show the presence/absence of 998 genes within 16 cyanosiphoviruses generated using Roary (Supplementary Data Sheet 1). Red color indicates the presence of gene, while the grey color shows that the gene is absent. X-axis indicates the 998 genes. The dendogram on the left and on the top of the heatmap was clustered using Bray-Curtis similarity based on the presence/absence of genes in genome.

**Figure S7** Dynamics of S-LBS1 (**A**) and *Synechococcus* *sp.* TCC793 (**B**) infected by S-LBS1 with different MOI. X-axis indicates the days after the infection. Each color indicates different MOI.

**3. Supplementary Data**

**Supplementary Data Sheet 1:** csv file shows genes presence/absence generated using Roary

**4. Supplementary References**

Angly, F. E., Felts, B., Breitbart, M., Salamon, P., Edwards, R. A., Carlson, C., et al*.* (2006). The marine viromes of four oceanic regions. PLoS Biol. 4, e368. doi: 10.1371/journal.pbio.0040368

Brum, J. R., Ignacio-Espinoza, J. C., Roux, S., Doulcier, G., Acinas, S. G., Alberti, A., et al*.* (2015) Ocean plankton. Patterns and ecological drivers of ocean viral communities. Science 348, 1261498. doi: 10.1126/science.1261498

Callieri, C., Coci, M., Corno, G., Macek, M., Modenutti, B., Beatriz, E., Bertoni, R. (2013). Phylogenetic diversity of non-marine picocyanobacteria. FEMS Microbiol. Ecol. 85, 293-301. doi: 10.1111/1574-6941.12118

Cai, L., Zhang, R., He, Y., Feng, X., Jiao, N. (2016). Metagenomic analysis of virioplankton of the subtropical Jiulong river estuary, China. Viruses 8: 35, doi: 10.3390/v8020035

Cellamare, M., Rolland, A., Jacquet, S. (2010). Flow cytometry sorting of freshwater phytoplankton. J. Appl. Phycol. 22, 87-100. doi: 10.1007/s10811-009-9439-4

Chan, Y. W., Millard, A. D., Wheatley, P. J., Holmes, A. B., Mohr, R., Whitworth, A. L., et al. (2015). Genomic and proteomic characterization of two novel siphovirus infecting the sedentary facultative epibiont cyanobacterium *Acaryochloris* marina. Environ. Microbiol*.* 17, 4239-52. doi: 10.1111/1462-2920.12735

Coloma, S. E., Dienstbier, A., Bamford, D. H., Sivonen, K., Roine, E., Hiltunen, T. (2017). Newly isolated *Nodularia* phage influences cyanobacterial community dynamics. Environ. Microbiol. 19, 273-86. doi: 10.1111/1462-2920.13601

Dillon, A., and Parry, J. D. (2008). Characterization of temperate cyanophages active against freshwater phycocyanin-rich *Synechococcus* species. Freshw. Biol. 43, 1253–1261. doi: 10.1111/j.1365-2427.2007.01938.x

Fancello, L., Trape, S., Robert, C., Boyer, M., Popgeorgiev, N., Raoult, D., et al*.* (2013). Viruses in the desert:a metagenomic survey of viral communities in four perennial ponds of the Mauritanian Sahara. ISME J*.* 7,359-369. doi: 10.1038/ismej.2012.101

Green, G. C., Rahman, F., Saxton, M. A., Williamson, K. E. (2015). Metagenomic assessment of viral diversity in Lake Matoaka, a temperate, eutrophic freshwater lake in southeastern Virginia, USA. Aquat. Microb. Ecol*.* 75, 117-128. doi: 10.3354/ame01752

Huang, S., Wang, K., Jiao, N., Chen, F. (2012). Genome sequences of siphoviruses infecting marine *Synechococcus* unveil a diverse cyanophage group and extensive phage-host genetic exchanges. Environ. Microbiol. 14, 540-558. doi: 10.1111/j.1462-2920.2011.02667.x

Hurwitz, B. L., and Sullivan, M. B. (2013). The Pacific Ocean Virome (POV): a marine viral metagenomic dataset and associated protein clusters for quantitative viral ecology. PLoS ONE 8, e57355. doi: 10.1371/journal.pone.0057355

Hurwitz, B. L., Westveld, A. H., Brum, J. R., Sullivan, M. B. (2014). Modeling ecological drivers in marine viral communities using comparative metagenomics and network analyses. Proc. Natl. Acad. Sci. USA. 111, 10714-9. doi: 10.1073/pnas.1319778111

Lopez-Bueno, A., Tamames, J., Velazquez, D., Moya, A., Quesada, A., Alcami, A. (2009). High diversity of the viral community from an Antarctic lake. Science 326, 858-861. doi: 10.1126/science.1179287

Mizuno, C. M., Rodriguez-Valera, F., Garcia-Heredia, I., Martin-Cuadrado, A. B., Ghai, R. (2013). Reconstruction of novel cyanobacterial siphovirus genomes from Mediterranean metagenomic fosmids. Appl. Environ. Microbiol. 79, 688-695. doi: 10.1371/journal.pgen.1003987

Personnic, S., Domaizon, I., Sime-Ngando, T., Jacquet, S. (2009). Seasonal variations of microbial abundances and virus- vs flagellate-induced mortality of picoplankton in three peri-alpine lakes. J. Plank. Res. 31, 1161-1177. doi: 10.1093/plankt/fbp057

Ponsero, A. J., Chen, F., Lennon, J. T., Wilhelm, S. W. (2013). Complete genome sequence of cyanobacterial siphovirus KBS2A. Genome Announcement. doi: 10.1128/genomeA.00472-13

Rodriguez-Brito, B., Li, L., Wegley, L., Furlan, M., Angly, F., Breitbart, M., et al*.* (2010). Viral and microbial community dynamics in four aquatic environments. ISME J*.* 4, 739-751. doi: 10.1038/ismej.2010.1

Rosario, K., Nilsson, C., Lim, Y. W., Ruan, Y., Breitbart, M. (2009). Metagenomic analysis of viruses in reclaimed water. Environ. Microbiol*.* 11, 2806-2820. doi: 10.1111/j.1462-2920.2009.01964.x

Roux, S., Enault, F., Robin, A., Ravet, V., Personnic, S., Theil, S., et al. (2012). Assessing the diversity and specificity of two freshwater viral communities through metagenomics. PLoS ONE 7, e33641. doi: 10.1371/journal.pone.0033641

Roux, S., Brum, J. R., Dutilh, B. E., Sunagawa, S., Duhaime, M. B., Loy, A., et al*.,* (2016). Ecogenomics and potential biogeochemical impacts of globally abundant ocean viruses. Nature 537, 689-93. doi: 10.1038/nature19366

Roux, S., Enault, F., Ravet, V., Colombet, J., Bettarel, Y., Auguet, J. C., et al*.,* (2016). Analysis of metagenomic data reveals common features of halophilic viral communities across continents. Environ. Microbiol*.* 18, 889-903. doi: 10.1111/1462-2920

Rusch, D. B., Halpern, A. L., Sutton, G., Heidelberg, K. B., Williamson, S., Yooseph, S., et al*.,* (2007). The Sorcerer II Global Ocean Sampling expedition: northwest Atlantic through eastern tropical Pacific. PLoS Biol. 5, e77. 10.1371/journal.pbio.0050077

Schoenfeld, T., Patterson, M., Richardson, P. M., Wommack, K. E., Young, M., Mead, D. (2008). Assembly of viral metagenomes from Yellowstone hot springs. Appl. Environ. Microbiol. 74, 4164-74. doi: 10.1128/AEM.02598-07

Six, C., Thomas, J. C., Brahamsha, B., Lemoine, Y. (2004). **Photophysiology of the marine cyanobacterium** Synechococcus **sp. WH8102, a new model organism**. Aquat. Microb. Ecol. 35, 17-29. doi: 10.3354/ame035017

Skvortsov, T., Leeuwe, C., Quinn, J. P., McGrath, J. W., Allen, C. C. R., McElarney, Y., et al*.* (2016). Metagenomic characterisation of the viral community of Lough Neagh, the Largest Freshwater Lake in Ireland. PLOS ONE 11, e0150361. doi: 10.1371/journal.pone.0150361

Sullivan, M. B., Krastins, B., Hughes, J. L., Kelly, L., Chase, M., Sarracino D., et al. (2009). The genome and structural proteome of an ocean siphovirus: a new window into the cyanobacterial « mobilome ». Environ. Microbiol*.* 11, 2935-2951. doi: 10.1111/j.1462-2920.2009.02081.x

Watkins, S. C., Kuehnle, N., Ruggeri, C. A., Malki, K., Bruder, K., Elayyan, J., et al*.* (2016). Assessment of a metaviromic dataset generated from nearshore Lake Michigan. *Mar.* Freshw. Res*.* 67, 1700-8. doi: 10.1071/MF15172

Zapata, M., Rodríguez, F., Garrido, J. L. (2000). Separation of chlorophylls and carotenoids from marine phytoplankton: A new HPLC method using a reversed phase C8 column and pyridine-containing mobile phases. Mar. Ecol. Prog. Ser*.* 195, 29-45. doi: 10.3354/meps195029
